# Interleukin-17A Facilitates Chikungunya Virus Infection by Inhibiting IFN-α2 Expression

**DOI:** 10.3389/fimmu.2020.588382

**Published:** 2020-11-16

**Authors:** Biswas Neupane, Dhiraj Acharya, Farzana Nazneen, Gabriel Gonzalez-Fernandez, Alex Sutton Flynt, Fengwei Bai

**Affiliations:** Department of Cell and Molecular Biology, Center for Molecular and Cellular Biosciences, The University of Southern Mississippi, Hattiesburg, MS, United States

**Keywords:** IL-17A, IFN-α2, chikungunya virus, IRF5/7, inflammation, mice

## Abstract

Interferons (IFNs) are the key components of innate immunity and are crucial for host defense against viral infections. Here, we report a novel role of interleukin-17A (IL-17A) in inhibiting IFN-α2 expression thus promoting chikungunya virus (CHIKV) infection. CHIKV infected IL-17A deficient (*Il17a^−/−^*) mice expressed a higher level of IFN-α2 and developed diminished viremia and milder footpad swelling in comparison to wild-type (WT) control mice, which was also recapitulated in IL-17A receptor-deficient (*Il17ra^−/−^*) mice. Interestingly, IL-17A selectively blocked IFN-α2 production during CHIKV, but not West Nile virus (WNV) or Zika virus (ZIKV), infections. Recombinant IL-17A treatment inhibited CHIKV-induced IFN-α2 expression and enhanced CHIKV replication in both human and mouse cells. We further found that IL-17A inhibited IFN-α2 production by modulating the expression of Interferon Regulatory Factor-5 (IRF-5), IRF-7, IFN-stimulated gene 49 (ISG-49), and Mx1 expression during CHIKV infection. Neutralization of IL-17A *in vitro* leads to the increase of the expression of these antiviral molecules and decrease of CHIKV replication. Collectively, these results suggest a novel function of IL-17A in inhibiting IFN-α2–mediated antiviral responses during CHIKV infection, which may have broad implications in viral infections and other inflammatory diseases.

## Introduction

Chikungunya virus (CHIKV) is a reemerging mosquito-transmitted alphavirus of the *Togaviridae* family that can cause crippling musculoskeletal inflammatory disease in humans that is characterized by fever, polyarthralgia, myalgia, rash, and headache. CHIKV, first isolated in 1953 in Tanzania, was endemic in the tropic regions of Africa and the Indian Ocean (1). Due to the increasing human travel and the rapid spread of the mosquito vectors to cooler climates, CHIKV has dramatically expanded its territory and is currently circulating in over 60 countries, including Africa, Asia, Europe, and the Americas (2). Since its introduction to the western hemisphere in 2013, CHIKV has caused an explosive epidemic in the Americas with over two million febrile cases with debilitating polyarthralgia in about 50 countries (3). However, there are no specific treatments or vaccines currently available, and the pathogenesis of CHIKV is largely unknown.

CHIKV is primarily transmitted to humans by the bites of infected female *Aedes aegypti* and *Aedes albopictus* mosquitos. After mosquito inoculation, CHIKV replicates in human epithelial cells, endothelial cells, and primary fibroblasts then enters the lymph nodes and finally disseminate to other tissues *via* the circulation (4, 5). Upon infection, the virus is sensed by diverse innate immune pattern recognition receptors (PRRs), such as toll-like receptors (TLRs), retinoic acid-inducible gene I (RIG I), melanoma differentiation-associated gene 5 (MDA5), and nod like receptors (NLRs). CHIKV infection induces the production of proinflammatory cytokines, chemokines, and type I interferons (IFN-α/β) (6). IFNα/β have been shown to play a key role in limiting CHIKV replication in both humans and mouse models (7). Fibroblasts are the primary target cells of CHIKV infection and also are the major producers of CHIKV-induced IFNα/β (8). Previous studies have revealed that the CHIKV infection induces antiviral responses by activating interferon regulatory factors (IRFs) (8, 9). IRF-5, along with IRF-3 and IRF-7 induces the Interferon stimulated genes (ISGs) during CHIKV infection (10). Further, IRF-7–mediated type I IFN responses provide critical antiviral protection as neonatal animals lacking either of these factors succumbed to CHIKV infection (8). Interestingly, CHIKV was one of the virus models used to discover and characterize the type I IFN (11–13). However, the regulation of type I IFN production and responses during CHIKV infection is still not clearly understood.

IL-17A, a best-characterized member of the IL-17 family, exerts diverse immune functions, including host defense from infection, tissue remodeling and repair, regulation of immune cell homing and inflammation, and cancer progression (14). For example, a high concentration of IL-17A in the joints contributes to inflammatory arthritis and other allergic and autoimmune diseases (15–20). IL-17A can facilitate WNV clearance from mouse brain by promoting CD8^+^ T-cell cytotoxicity (21) and also inhibit the replication of HSV by enhancing IFN-γ^+^ Th1 cell response (22). IL-17A signaling also has a protective role to the host during infection of adenovirus by inducing high levels of IL-7R and RORγt expression in mouse liver cells (23, 24). Similarly, IL-17A has also been suggested to facilitate the infection of coxsackievirus B3 by decreasing splenic CD8^+^ T cell numbers and cardiac IFN-γ production (25) and Theiler’s murine encephalomyelitis virus by up-regulating anti-apoptotic molecules (26, 27). During CHIKV infection, the serum IL-17A levels in patients with arthritis were higher than non-arthritic patients (28). In addition, strong associations between IL-17A levels and swollen joints have been identified (28, 29), suggesting IL-17A might play an important role in the pathogenesis of CHIKV; however, its detailed role has not been characterized. In this study, we identified a novel function of IL-17A signaling in promoting CHIKV infection by negatively regulating IFN-α2–mediated antiviral responses.

## Materials and Methods

### Ethics Statement and Biosafety

All the experimental procedures involving animals in this study were reviewed and approved by the Institutional Animal Care and Use Committees at The University of Southern Mississippi (USM). Experiments and animal studies involving live CHIKV were performed by certified personnel in Biosafety Level 3 (BSL-3) laboratories following the biosafety protocols approved by the USM Institutional Biosafety Committee.

### Viruses

CHIKV (LR OPY1 2006 strain) was provided by University of Texas Medical Branch. A single passage of parental viruses was propagated in Vero cells (ATCC CCL-81) and used as viral stock for this study. The viral stocks were titrated in Vero cells by plaque assay as previously described (30, 31).

### Cells

Vero cells, NIH3T3 cells (ATCC CRL-1658), and Raw 264.7 cells (ATCC TIB-71) were maintained in Dulbecco’s modified Eagle’s medium (DMEM, Life Technologies) containing 1% L-glutamine, 1% penicillin/streptomycin, and 10% fetal bovine serum (FBS). Saos2 cells (ATCC HTB-85) were cultured in McCoy’s 5A medium (ATCC 30-2007) supplemented with 15% FBS. All cells were kept in an incubator at 37°C with 5% CO_2_, and relative humidity of about 95%. Mouse BMDMs and BMDCs were prepared according to previous publications (32–34).

### Mice

WT C57BL/6J mice were purchased from the Jackson Laboratory (Bar Harbor, ME). Breeding pairs of *Il17a^−/−^* and *Il17ra^−/−^* mice (both in a C57BL/6J background) were provided by Richard A. Flavell (Yale University School of Medicine) and Dr. Sarah Gaffen (University of Pittsburgh), respectively. All the breeders and their pups were kept in a pathogen-free room. Viral infection studies were carried out in the BSL-3 animal facility at USM.

Seven to eight weeks old, sex-matched WT, *Il17a^−/−^ and Il17ra^−/−^* mice were subcutaneously injected on the ventral side of the left hind footpad toward the ankle with 1 × 10^5^ PFU of CHIKV in 50-µl phosphate buffer saline (PBS), as mentioned in the previous publications (33, 35). Starting from the day of infection (day 0) to 12 days p.i., the thickness and width of the peri-metatarsal area of the infected feet were measured daily using a digital caliper (Electron Microscopy Science). The relative increase in footpad swelling was calculated and expressed in comparison to pre-infection as previously described (33–36). Blood samples were collected in 0.5M EDTA by retro-orbital bleeding to analyze viral RNA and cytokines. Mice were sacrificed at the selected time-points for collection of the infected footpad.

### Quantitative PCR

Total RNA was converted into the first-strand complementary DNA (cDNA) using iSCRIPT™ cDNA synthesis kit (Bio-Rad). QPCR assays were performed in a CFX Connect Real-Time System (Bio-Rad) using SYBR Green supermix (Bio-Rad) for the detection of *CHIKV-E1*, immune response genes, and *β-actin*. Viral RNA copy numbers were expressed as the ratio of *CHIKV-E1* to cellular *β-actin*. For QPCR assay of immune response genes, data were expressed as relative fold change (RFC) expressed by ^ΔΔ^CT method after normalizing to cellular *β-actin*. CHIKV-E1 and cytokine transcripts were also quantified by RT-QPCR in total RNA extracted from footpad cells. The primer sequences were designed using NCBI’s primer-designing tool and synthesized by Integrated DNA Technologies or Invitrogen (**Supplementary Table 1**).

### Plaque Assay

Vero cells were plated in 6-welled plates at a density of 6 × 10^5^ cells per well and incubated overnight. Cell culture supernatant or mouse serum samples were applied to the wells and incubated for 1 h at 37°C with 5% CO_2_. After virus adsorption, the inoculum was removed, and the cells were covered with an overlay medium containing 1% SeaPlaque agarose (Lonza). The plates were incubated for 2 to 3 days for plaque development. Plaques were visualized by staining with Neutral Red and counted.

### Flow Cytometry

For flow cytometric analysis of footpad immune cells, the infected footpad tissues were collected to prepare single-cell suspension as previously described protocol (37). Briefly, the infected footpads were chopped into small pieces and incubated for 1 h at 37°C in digestion medium containing hyaluronidase and collagenase type VIII. The footpad cells were collected after filtering the mixture with 70-µm strainer. The footpad cells were fixed in 2% paraformaldehyde (Electron Microscopy Science) for 15 min at room temperature (RT). The cells were then washed and blocked with Fc block for 30 min at RT. After washing, the cells were probed with mouse monoclonal anti-CD45, CD4, CD8, CD11b, and Ly6G antibodies (BD BioSciences or eBioscience) and incubated for 1 h at RT. The cells were then washed twice and resuspended in staining buffer and analyzed in a BD LSRFortessa (BD Biosciences) using FlowJo (Version 10.4).

### Histology

The infected and control mouse footpads were collected on day 6 p.i. After fixation overnight in 4% PFA, the footpads were decalcified in 10% EDTA for 10 days. Tissues were then dehydrated; paraffin embedded, and sectioned (10 µm) using a microtome (American Optical Spencer 820). The sectioned tissue slides were stained with hematoxylin and eosin (H&E), and images were acquired using a bright-field microscope (Olympus BH2).

### ELISA and Immunoblotting

IL-17A and IFN-α2 in the cell culture supernatants of CHIKV-infected NIH3T3 cells was measured by using a commercial ELISA kit (Abcam) following the manufacturer’s instruction. NIH3T3 cells were infected with CHIKV at 1 MOI in the presence of recombinant IL-17A. After 24 h, the cells were lysed in Laemmli sample buffer (Bio-Rad), and the proteins were separated by 10% SDS-polyacrylamide gel electrophoresis, transferred to a nitrocellulose membrane (Bio-Rad). The membrane was blocked in 5% milk in Tris-buffered saline with Tween 20 (TBS-T) for 1h at RT and probed with mouse specific Rabbit primary antibody (Phospho-IRF-7, Cell Signaling; Phospho-IRF-5, Invitrogen; GAPDH, Abcam) in the ratio 1:1,000 at 4°C overnight in a rocker. After washing with TBS-T, horseradish peroxidase conjugated secondary antibody (Goat pAb to Rabbit IgG, Abcam; 1:5,000) was added for 1 h at RT. The membranes were washed and developed using SuperSignal West Pico Chemiluminiscence Substrate (Thermo Scientific) and images were acquired using a ChemiDoc MP system (Bio-Rad). Quantification of blot was performed by ImageLab.

### Statistical Analyses

Data analysis was performed by using either a Student’s t-test or one-way analysis of variance (ANOVA) wherever applicable in GraphPad Prism software (version 6.0). *p* < 0.05 was considered statistically significant.

## Results

### CHIKV Induces the Expression of *Il-17a* *In Vitro* and *In Vivo*

**Figure 1:**
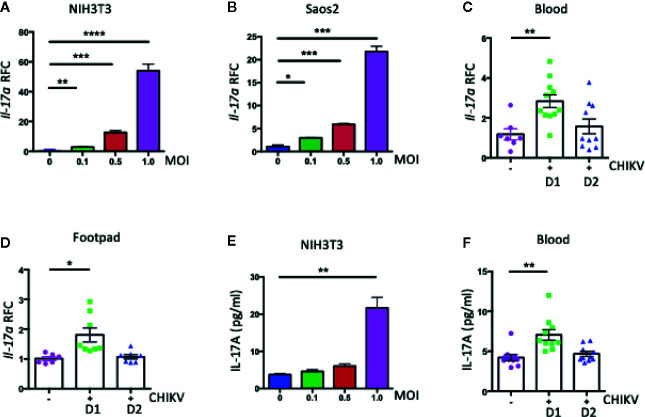
CHIKV infection induces the expression of *Il17a* in mouse and human. **(A, B)** The expression of *Il17a* transcripts was measured by QPCR in NIH3T3 **(A)** and Saos2 **(B)** cells that were infected with indicated MOIs of CHIKV for 24 h and expressed as Relative Fold Changes (RFC) after normalization to cellular *β-actin* transcripts. WT (C57BL/6J) mice (7 to 8 weeks old) were inoculated with CHIKV (1 × 10^5^ PFU) or vehicle control (PBS) *via* footpad, and blood was collected on days **(D)** 1 and 2 post-infection (p.i.). **(C, D)** RFC in the expression of *Il17a* transcripts in blood **(C)** and footpad **(D)** of CHIKV-infected mice was compared with that of mock-infected control mice after normalization to *β-actin*. **(E, F)** The protein levels of IL-17A was measured by ELISA in NIH3T3 cells **(E)** and mice blood **(F)**. The results were analyzed either by using one-way ANOVA followed by Tukey’s test **(A, B, E)** or a two-tailed student’s *t*-test **(C, D, F)**. The results are representative of at least two independent experiments and are expressed as mean ± the standard errors of the mean (SEM) [n = 3 biological replicates for **(A, B, E)**; n = 7 to 11 mice/group for **(C, D, F)**. *, **, ***, and **** denote *p* < 0.05, *p* < 0.01, *p* < 0.001, and *p* < 0.0001, respectively.

IL-17A is a proinflammatory cytokine that plays essential roles in infections and inflammatory diseases. To test our hypothesis if IL-17A plays a role in CHIKV pathogenesis, we measured its expression in CHIKV-infected mouse and human cells. Mouse fibroblasts (NIH3T3 cells) and human bone epithelial cells (Saos2 cells) were infected with CHIKV (MOI 0.1, 0.5, and 1) for 24 h, and transcripts of *Il-17a* and cellular *β-actin* (a housekeeping gene) were measured by a reverse-transcription quantitative PCR (QPCR). The results showed an upregulation of *Il-17a* transcripts by CHIKV in both NIH3T3 cells (**Figure 1A**) and Saos2 cells (**Figure 1B**) in a dose-dependent manner. To relate these results in a mouse model, we infected C57BL/6J wild-type (WT) mice with CHIKV *via* footpad inoculation, and the blood and footpad samples were collected on days (D) 1 and 2 post-infection (p.i.). Similar to the *in vitro* results, *Il-17a* transcript levels were significantly upregulated in the blood and footpads of CHIKV-infected mice at D1 p.i. (**Figures 1C, D**). In addition, ELISA results showed significant increases of IL-17A at protein levels in the NIH3T3 cell culture (**Figure 1E**) and the plasma samples on D1 p.i. (**Figure 1F**). These results demonstrate that CHIKV infection induces the expression of IL-17A in physiologically relevant human and mouse cells and mice.

### IL-17A Promotes CHIKV-Induced Footpad Swelling in Mice

**Figure 2:**
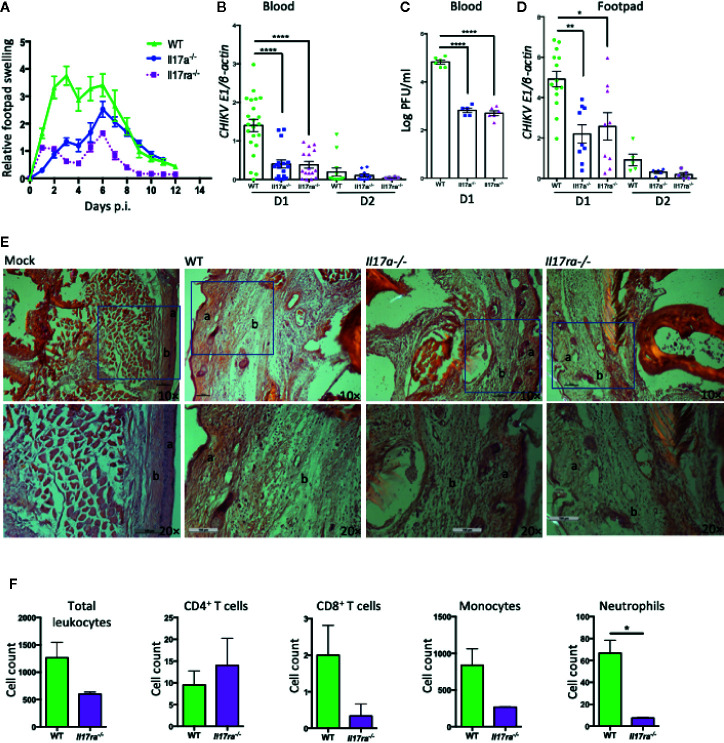
*Il17a^−/−^* and *Il17ra^−/−^* mice are more resistant to CHIKV infection. Seven to eight weeks old WT, *Il17a^−/−^* and *Il17ra^−/−^* mice were infected with 1 × 10^5^ PFU of CHIKV *via* footpad. **(A)** Swelling of injected footpads (peri-metatarsal area) of mice is presented as a relative increase in swelling that was calculated by measuring the thickness and width of an inoculated footpad. **(B)** Blood was collected on days 1 (D1) and D2 p.i., and QPCR was performed to measure the copies of *CHIKV E1* and cellular *β-actin* transcripts. Viral burden was expressed as a ratio of copy number of *CHIKV E1* to cellular *β-actin* transcripts and compared between WT (n = 22) and *Il17a^−/−^* (n = 17) or *Il17ra^−/−^* (n = 16) mice. **(C)** Plaque assay was performed to measure the viral load in the blood samples of D1 from **(B)**. **(D)** Inoculated footpads of WT (n = 14) and *Il17a^−/−^* (n = 9) or *Il17ra^−/−^* (n = 9) were collected on D1 and D2 p.i., and the viral burden was quantified by QPCR after normalization to cellular *β-actin*. **(E)** Representative H&E stained histological images (10×, upper row and 20×, lower row) of peri-metatarsal area of the inoculated foot tissue (n = 5 mice/group) on D6 p.i. The layer of epidermis (a) and dermis (b) are labeled. **(F)** Seven to 8 weeks old WT (n = 4) and *Il17ra^−/−^* mice (n = 3) were infected with 1 × 10^5^ PFU of CHIKV *via* footpad. Footpad cells were isolated at D6 p.i., and quantified by flow cytometry after probing with antibodies against CD45, CD4, CD8, CD11b, and Ly6G. The cell counts for positive cells within the gated population are shown for WT and *Il17ra^−/−^* mice. The results are expressed as mean ± SEM **(A**–**D**, **F)** or a representative image **(E)** that represents at least three independent experiments. The data were analyzed by a two-tailed Student t-test **(A**–**D**, **F)**. *, **, and **** denote *p* < 0.05, *p* < 0.01, and *p* < 0.0001 respectively, when compared to WT controls).

To investigate the role of IL-17A in CHIKV-induced disease, we infected 7 to 8 weeks old, sex-matched WT, IL-17A–deficient (*Il17a^−/−^*), and IL-17A receptor-deficient (*Il17ra^−/−^*) mice with 1 × 10^5^ PFU of CHIKV *via* footpad, as described in the previous reports (32, 37). Following infection, the CHIKV inoculated footpads usually swell between D1 and D12 p.i. in the adult WT C57BL/6J mice (32, 38). We, therefore, monitored footpad swelling daily for up to 12 days and calculated the relative increase in footpad swelling of CHIKV-infected WT, *Il17a^−/−^* and *Il17ra^−/−^* mice. The swelling data indicated that *Il17a^−/−^* and *Il17ra^−/−^* mice had milder inflammation in their inoculated footpads compared to WT control mice (**Figure 2A**). To corroborate these observations, we collected blood on D1 and 2 p.i. and the viremia was measured by both QPCR and plaque assay. The QPCR results showed a three-fold mean reduction of *CHIKVE1* transcripts on D1 p.i. in the *Il17a^−/−^* and *Il17ra^−/−^* mice compared to WT mice (**Figure 2B**), whereas the CHIKV was mostly cleared from the blood on D2 p.i. Consistent with QPCR, the plaque assay also showed less infectious viruses in both *Il17a^−/−^* and *Il17ra^−/−^* mice (**Figure 2C**). After inoculation, the CHIKV initially replicates locally in the footpad tissues. Therefore, to assess whether the systemic viral replication is similar to viral replication in the infected footpad, we sacrificed selected mice and collected the tissues of the infected footpad on D1 and 2 p.i. and performed the QPCR analysis. Consistent with the viremia, there was an approximately two-fold reduction in *CHIKVE1* RNA in the foot tissues of *Il17a^−/−^* and *Il17ra^−/−^* mice compared to the WT controls on D1 (**Figure 2D**). Despite the differences are not statistically significant, a similar trend remains on D2 p.i. (**Figure 2D**).

To further dissect this observation, we performed histological and flow cytometric analysis with the inoculated footpad tissues at peak swelling (D6 p.i.). The histological analysis showed that the peri-metatarsal area of the inoculated footpads of *Il17a^−/−^* and *Il17ra^−/−^* mice had less severe swelling and tissue damage compared to that of WT mice (**Figure 2E**). In addition, the flow cytometric analysis demonstrated a significantly reduced infiltration of neutrophils (CD45^+^CD11b^+^Ly6G^+^) in the footpad of *Il17ra^−/−^* mice compared to WT mice (*p* < 0.05), while the differences are not statistically significant (*p* > 0.05) for other immune cells including CD4 T cells (CD45^+^CD3^+^CD4^+^), CD8 T cells (CD45^+^CD3^+^CD8^+^), and monocytes (CD45^+^CD11b^+^) (**Figure 2F**). A similar trend, but with no statistical difference, of these infiltrated cells was also observed in *Il17a^−/−^* mice compared to WT mice (data not shown). Together, these results suggest that IL-17A signaling facilitates CHIKV replication and exacerbates CHIKV-induced footpad inflammation in mice.

### IL-17A Inhibits the *Ifn-α2* Expression to Promote CHIKV Replication

**Figure 3:**
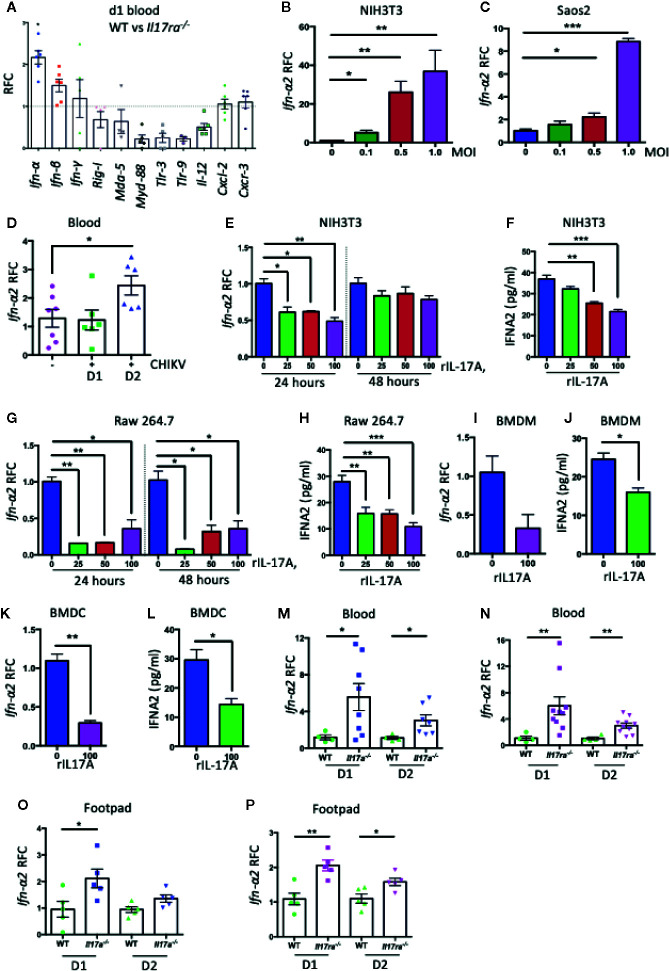
IL-17A down-regulates the expression of *Ifn-α2.* **(A)** Seven to eight weeks old WT (n = 5) and *Il17ra^−/−^* mice (n = 6) were infected with 1 × 10^5^ PFU of CHIKV *via* footpad and the transcripts of indicated genes in blood were measured at day 1 post-infection by QPCR and normalized to *β-actin* mRNA. **(B)** NIH3T3 and **(C)** Saos2 cells were infected with CHIKV for 24 h, and the transcript level of *Ifn-α2* was measured by QPCR and normalized to *β-actin* mRNA. **(D)** Seven to eight weeks old WT mice were mock infected (n = 7) or infected with 1 × 10^5^ PFU of CHIKV (n = 6) *via* footpad and the expression of *Ifn-α2* was measured by QPCR in the blood at indicated time points and normalized to *β-actin* mRNA. **(E)** NIH3T3 cells were infected with CHIKV (MOI = 1) and treated with indicated concentrations of rIL-17A for 24 and 48 h. The expression of *Ifn-α2* was measured by QPCR and normalized to cellular *β-actin* mRNA. **(F)** The protein level of IFN-α2 was measured by ELISA in NIH3T3 cells infected with CHIKV (MOI = 1) and treated with different concentrations of rIL-17A. **(G, H)** Raw 264.7 cells were infected with CHIKV (MOI = 1) in the presence of indicated concentrations of rIL-17A and incubated for 24 and 48 h followed by the measurement of mRNA level **(G)** and protein level **(H)** of IFN-α2. **(I**–**L)** Mouse bone marrow derived-macrophages **(I, J)** and dendritic cells **(K**, **L)** were infected with CHIKV (MOI = 1) for 48 h in the presence of rIL-17A (100 ng/ml). The level of *Ifn-α2* was measured either by QPCR normalized to cellular *β-actin* mRNA or by ELISA. **(M**, **N)** WT (n = 4), *Il17a^−/−^* (n = 8) and *Il17ra^−/−^* mice (n = 10) were infected with 1 × 10^5^ PFU of CHIKV *via* footpad. Blood was collected on days 1 and 2 post-infection, and the mRNA level of *Ifn-α2* was measured by QPCR and normalized to *β-actin* mRNA between WT and *Il17a^−/−^* mice **(M)**, and WT and *Il17ra^−/−^* mice **(N)**. *Ifn-α2* mRNA levels in the footpads of *Il17a^−/−^* mice **(O)** and *Il17ra^−/−^* mice **(P)** were measured by QPCR and normalized to *β-actin* mRNA. All the data represent at least two independent experiments performed in triplicates and analyzed by one-way ANOVA followed by Turkey’s test, (*, **, and *** denote *p* < 0.05, *p* < 0.01, and *p* < 0.001 respectively, when compared to untreated control).

As we observed that the deficiency in IL-17A signaling in mice led to a significantly reduced CHIKV replication in the blood and footpad tissues, we hypothesized that IL-17A signaling may inhibit the antiviral innate immune responses. To test this, we profiled the expression of a set of innate antiviral genes in CHIKV-infected WT and *Il17ra^−/−^* mice by QPCR analysis. Interestingly, we found that among 11 different genes tested (*Ifn-α*, *Ifn-β*, *Ifn-*γ**, *Rig-I*, *Mda-5*, *Myd88*, *Tlr3*, *Tlr9*, *Il-12*, *Cxcl2*, *and Cxcr3*), the expression of *Ifn-α* was significantly increased in the blood of *Il17ra^−/−^* mice (**Figure 3A**). There are multiple *Ifn-α* subtypes in mice, and we further tested the effect of IL-17A signaling in the expression of these subtypes upon CHIKV infection. Interestingly, of the 11 *Ifn-α* subtypes tested, specifically the expression of *Ifn-α2* transcripts was significantly inhibited in CHIKV-infected NIH3T3 cells (**Supplementary Figure 1**). To further validate these results, we first confirmed that the expression of *Ifn-α2* was induced by CHIKV in NIH3T3, Saos2 cells, and mice (**Figures 3B–D**). We next tested if the expression of CHIKV-induced *Ifn-α2* in NIH3T3 cells could be inhibited by recombinant mouse IL-17A (rIL-17A). Both QPCR and ELISA results indicated that rIL-17A treatment suppressed the expression of CHIKV-induced *Ifn-α2* (**Figures 3E, F**) in NIH3T3 cells. Consistently, this phenotype was also confirmed in other cell types, i.e., Raw 264.7 cells, mouse bone marrow–derived macrophages (BMDM), and bone marrow–derived dendritic cells (BMDCs) (**Figures 3G–L**), suggesting a cell type-independent effect. We next measured the expression levels of *Ifn-α2* in *Il17a^−/−^* and *Il17ra^−/−^* mice that lack IL-17A signaling to further determine if IL-17A signaling inhibits the expression of *Ifn-α2* *in vivo* following CHIKV infection. Indeed, the mRNA level of *Ifn-α2* in blood and footpads was increased in both *Il17a^−/−^* and *Il17ra^−/−^* mice than in WT mice, confirming the inhibitory effect of IL-17A on the CHIKV-induced expression of *Ifn-α2* (**Figures 3M–P**).

**Figure 4:**
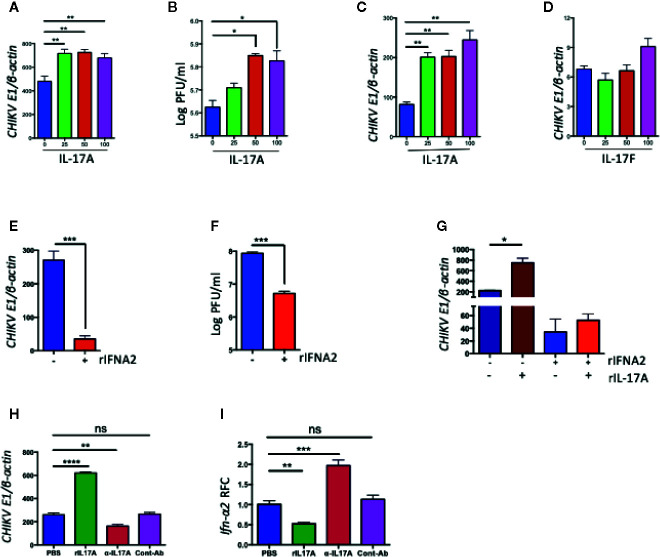
IL-17A, but not IL-17F, promotes CHIKV replication in NIH3T3 cells. **(A, B)** NIH3T3 cells were infected with CHIKV (MOI =1) in the presence of indicated concentrations of rIL-17A for 48 h. The ratio of *CHIKVE1* to cellular *β-actin* transcripts was determined by QPCR **(A)** and the number of infectious virus in the culture supernatants were measured by a plaque assay **(B)**. **(C)** NIH3T3 cells were preincubated for 24 h with indicated concentrations of rIL-17A, then infected with CHIKV (MOI = 1) for an additional 48 h. The ratio of *CHIKVE1* RNA to cellular *β-actin* transcripts was determined by QPCR. **(D)** NIH3T3 cells were infected with CHIKV (MOI = 1) in the presence of IL-17F for 48 h, and the ratio of *CHIKVE1* RNA to cellular *β-actin* transcripts was determined by QPCR. **(E, F)** NIH3T3 cells were treated with recombinant IFNA2 for 8 h and then infected with CHIKV at 1 MOI for 24 h. The ratio of *CHIKVE1* to cellular *β-actin* transcripts was determined by QPCR **(E)** and the infectious virus in the cell medium were measured by a plaque assay **(F)**. **(G)** NIH3T3 cells were treated with rIL-17A and IFNA2, then infected with CHIKV at 1 MOI for 24 h. The ratio of *CHIKVE1* to cellular *β-actin* transcripts was measured by QPCR. **(H, I)** NIH3T3 cells were infected with CHIKV (1 MOI) in the presence of rIL-17A, IL-17A neutralizing antibody or anti-flavivirus 4G2 antibody as an isotype control for 24 h. The ratio of *CHIKVE1* to cellular *β-actin* mRNA was determined by QPCR **(H)** and the expression of *Ifn-α2* was measured by QPCR and normalized to cellular *β-actin* mRNA **(I)**. All the data represent at least two independent experiments performed in triplicates and analyzed by one-way ANOVA followed by Turkey’s test, (*, **, and *** denote *p* < 0.05, *p* < 0.01, and *p* < 0.001, respectively, when compared to untreated control). ns denotes not significant.

To test if IL-17A also inhibits the expression of *Ifn-α2* during other virus infections, we infected NIH3T3 cells with West Nile virus (WNV) or Zika virus (ZIKV) in the presence of rIL-17A, and measured *Ifn-α2* mRNA by QPCR or ELISA. Interestingly, the results suggested that IL-17A has no such effect on IFN-α2 production during WNV and ZIKV infections (**Supplementary Figures 2A–C**). IL-17F, a closely related member of IL-17 family, has related functions to IL-17A and also shares the same set of receptors IL-17RA and IL-17RC. To test if IL-17F function similarly with IL-17A in the regulation of IFN-α2, we measured the level of CHIKV-induced *Ifn-α2* expression in NIH3T3 cells upon recombinant IL-17F (rIL-17F) treatment (**Supplementary Figures 2D, E**). Our results demonstrated that only IL-17A, but not IL-17F, inhibited the CHIKV-induced IFN-α2 expression. As a consequence, the treatment of rIL-17A after CHIKV infection significantly increases the virus replication in NIH3T3 cells (**Figures 4A, B**), which may be due to the reduced antiviral IFN-α2 production. In another experiment, we also tested if the cells pretreated with rIL-17A could enhance susceptibility to CHIKV infection. The results showed that the pretreatment with rIL-17A, but not rIL-17F, enhanced CHIKV replication (**Figures 4C, D**), implying that IL-17A constrains the production of IFN-α2 thus facilitating CHIKV replication. The antiviral activities of IFN-α2 were confirmed by the experiment showing that the presence of rIFN-α2 constrains CHIKV replication in NIH3T3 cells (**Figures 4E, F**), while the addition of rIL-17A neutralizes the antiviral effects of rIFN-α2 (**Figure 4G**). However, it does not rule out that the observed effect of IL-17A happens independently of CHIKV infection. Next, we tested if the presence of IL-17A neutralizing antibody could inhibit the effects of CHIKV-induced IL-17A. We infected NIH3T3 cells with CHIKV in the presence of IL-17A neutralizing antibody or anti-flavivirus 4G2 antibody as an isotype control. The results show that CHIKV replication is inhibited and the expression level of *Ifn-α2* increases in the presence of IL-17A neutralizing antibody, but not of the control antibody (**Figures 4H, I**). Collectively, these results demonstrate that IL-17A signaling inhibits the production of IFN-α2, thus facilitating the CHIKV replication *in vitro* and mice.

### IL-17A Inhibits the Expression of *Ifn-α2 via* IRF-5 and 7 Pathways

**Figure 5:**
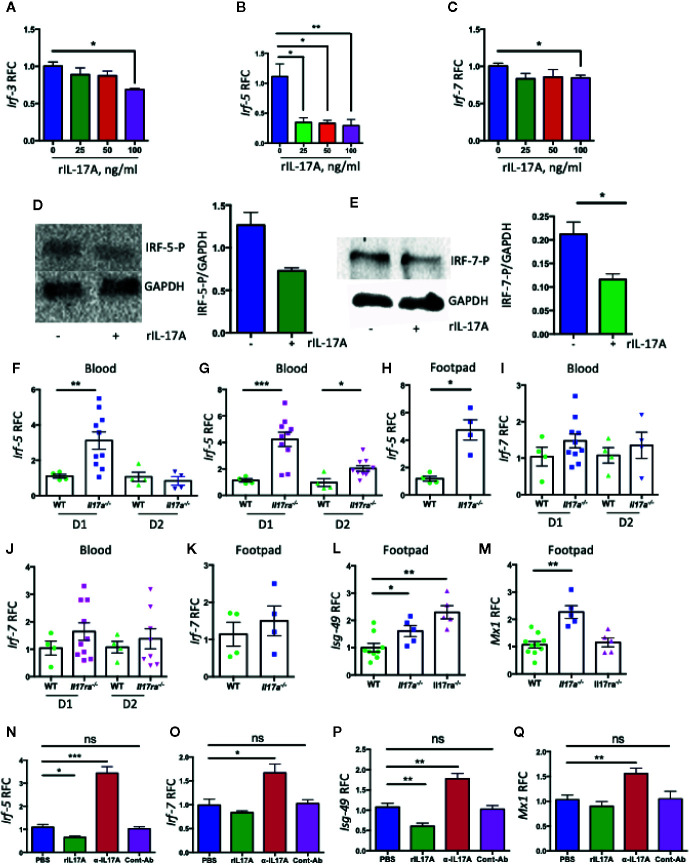
IL-17A downregulates the expression of *Irf5* and *Irf7* during CHIKV infection. NIH3T3 cells were infected with CHIKV (MOI = 1) and simultaneously treated with indicated concentrations of rIL-17A for 48 h. The transcripts of *Irf-3* **(A)**, *Irf-5* **(B)**, and *Irf-7* **(C)** were measured by QPCR and expressed as RFC after normalization to cellular *β-actin* mRNA. **(D)** NIH3T3 cells were infected with CHIKV (MOI = 1) for 24 h in the presence of IL-17A (100 ng/ml) and indicated proteins in the cell lysates were analyzed by immunoblotting (left) and the ratio of phosphorylated IRF-5 to GAPDH was calculated by densitometric measurement by using ImageLab (right). **(E)** The immunoblotting analysis was done similarly for IRF-7 and GAPDH (left), and the ratio of phosphorylated IRF-7 to GAPDH was calculated by densitometric measurement by using ImageLab (right). **(F, G)** WT (n = 4), *Il17a^−/−^* (n = 10), and *Il17ra^−/−^* (n = 10) mice were infected with 1 × 10^5^ PFU of CHIKV *via* footpad. Blood was collected on days 1 and 2 post-infection, and the mRNA levels of *Irf-5* were measured and normalized to *β-actin* mRNA between WT and *Il17a^−/−^* mice **(F)**, and WT and *Il17ra^−/−^* mice **(G)**. Footpads were collected from selected WT (n = 4) and *Il17a^−/−^* (n = 4) mice on D1 p.i., and the mRNA level of *Irf-5* was measured and normalized to *β-actin* mRNA between WT and *Il17a^−/−^* mice **(H)**. The mRNA levels of *Irf-7* were measured in the blood of *Il17a^−/−^* mice **(I)**, the blood of *Il17ra^−/−^* mice **(J)**, and the footpads of *Il17a^−/−^* mice **(K)**. **(L,** **M)** *Isg-49* and *Mx1* mRNAs in the footpads (n = 5 to 10/group) on D1 p.i. **(N**–**Q)** NIH3T3 cells were infected with CHIKV (1 MOI) in the presence of IL-17A, anti-Il17A antibody or 4G2 control antibody for 24 h. The level of *Irf-5* (N), *Irf-7* **(O)**, *Isg-49* **(P)**, and *Mx1* **(Q)** were measured and normalized to cellular *β-actin* mRNA. The data **(A**–**C** and **N**–**Q)** represent the results of two independent experiments carried out in triplicates, and analyzed by one-way ANOVA followed by Turkey’s test, (*, **, and *** denote *p* < 0.05, *p* < 0.01, and *p* < 0.001 respectively, when compared to untreated control). The results **(F**–**M)** represent three independent experiments analyzed by a two-tailed Student t-test, (*, **, and *** denote *p* < 0.05, *p* < 0.01, and *p* < 0.001 respectively, when compared to WT controls). ns denotes not significant.

We next hypothesized that IL-17A signaling interrupts the signaling pathway(s) that differentially regulate the expression of *Ifn-α* subtypes and thus inhibiting the expression of *Ifn-α2*. Relative amounts of IRF genes have been shown to modulate the differential expression of various IFN-A subtypes during paramyxovirus infection (39). IRF genes have been shown to play protective roles against CHIKV infection (9). Therefore, we assessed the role of IL-17A signaling in the expression of IRF genes during CHIKV infection. We infected NIH3T3 cells with CHIKV in the presence of rIL-17A for QPCR analysis, and the results showed that the expression of *Irf-3*, *Irf-5*, and *Irf-7* was inhibited at the transcriptional levels (**Figures 5A–C**). In addition, the immunoblotting analysis suggests that IL-17A inhibits the phosphorylation IRF-5 and IRF-7 during CHIKV infection (**Figures 5D, E**), while the phosphorylation of IRF-3 was not detected (data not shown). Considering IL-17A inhibits the expression and phosphorylation of IRF-5 and IRF-7 in the cell culture during CHIKV infection, we measured the levels of *Irf-5* and *Irf-7* mRNA in mice blood and footpads following CHIKV infection. Consistent with the results, the levels of *Irf-5* are significantly upregulated in the blood and footpads of *Il17a^−/−^* and *Il17ra^−/−^* mice compared to WT control mice (**Figures 5F–H**). A similar trend is also shown for *Irf-7*, although the difference is not statistically significant (**Figures 5I–K**). These results indicate that IL-17A may inhibit the expression of *Ifn-*α2 *via* IRF-5 and 7 pathways. We next tested if IL-17A signaling inhibits IFN-mediated antiviral responses. We tested the expression of a set of ISGs (*Isg-15*, *Isg-49*, *Isg-54*, *Isg-56*, *Oas1a*, *Mx1*, *Ifitm3*, and *bst2*) by QPCR in the blood and footpads of WT, *Il17a^−/−^* and *Il17ra^−/−^* mice following CHIKV infection. Interestingly, we found that among those test ISGs, *Isg-49*, and *Mx1* were significantly upregulated in *Il17a^−/−^* and *Il17ra^−/−^* mice footpads compared to the WT controls (**Figures 5L–M**). Similar patterns were observed in the blood, although the difference was not significant (data not are shown). We further confirmed that the expression of *Irf-5*, *Irf-7*, *Isg-49*, and *Mx1* were upregulated in the presence of IL-17A neutralizing antibody in CHIKV-infected NIH3T3 cells (**Figures 5N–Q**). Collectively, these results demonstrate that IL-17A signaling inhibits IFN-α2–mediated antiviral responses during CHIKV infection.

## Discussion

IL-17A signaling has been associated with several inflammatory diseases in humans, such as rheumatoid arthritis (40, 41), systemic lupus erythematosus (42), and Crohn’s disease (43, 44). Among its diverse functions, IL-17A can regulate the activities of various other inflammatory cytokines, which include TNF-α, IL-1β, and IFN-γ (45–48). For example, IL-17A signaling has also been shown to upregulate joint destructive factors by stimulating transcriptional NF-κB activity and expression of IL-1, granulocyte/macrophage colony-stimulating factor (GM-CSF), prostaglandin E2, IL-6, IL-8, and TNF-α in fibroblasts, endothelial and epithelial cells, and inducing T cell proliferation (46, 49–56). In this study, we report a novel role of IL-17A in inhibiting IFN-α2 expression during CHIKV infection.

CHIKV infection in humans can induce severe inflammatory responses and chronic arthritis, which is correlated with the increased production of IL-17A in patients (6, 57). As such, it is plausible to hypothesize that IL-17A may play an important role in the pathogenesis of CHIKV. We detected a significant up-regulation of *Il-17a* in both human and mouse cells, and mouse blood and footpads following CHIKV infection (**Figure 1**). Interestingly, the CHIKV burden was negatively regulated by IL-17A signaling as both *Il17a^−/−^* and *Il17ra^−/−^* mice exhibited lower CHIKV loads in their blood and mild swelling in inoculated footpads compared to those of WT mice (**Figure 2**). These results suggest that IL-17A signaling facilitates CHIKV replication and disease in mice. In mouse models, after subcutaneous inoculation of CHIKV *via* footpad, biphasic swelling responses usually occur in the inoculated footpad. Two peaks of swelling can be observed on around days 1–3 and 5–7 p.i., respectively. Mice also develop severe arthritis, tendonitis, and fasciitis in the inoculated foot, but these inflammatory effects are generally milder in the contralateral foot without swelling (32, 34, 38). Consistent with these results, in our study, the major peak of CHIKV-induced footpad swelling occurred on day 6 p.i. when the viruses had been cleared from the animal circulation, which suggests that the second peak of the swelling is largely mediated by pathogenic immune responses.

Type I IFN signaling has been shown to play an essential role in controlling infections of alphaviruses including CHIKV (58–60). Inhibition of type I IFN signaling in mice causes severe CHIKV-associated disease due to higher viral loads and virus dissemination to the CNS (58, 61). As the major target cells, fibroblasts can be infected by CHIKV *in vitro* and *in vivo* (4, 7) and are the major source of type I IFNs responding to CHIKV infection (7). Our results on NIH3T3 cells indicate that IL-17A inhibits IFN-α2 expression during CHIKV infection (**Supplementary Figure 1** and **Figure 3**). We also showed the same effect of IL-17A on the other cell types, such as BMDMs and dendritic cells, as well as mouse macrophage cells (Raw 264.7), suggesting that IL-17A inhibits IFN-α2 expression during CHIKV infection in a cell-type independent manner. The blockade of IFN-α in mice results in the increases of CHIKV viral loads in the infected foot and serum demonstrating its importance in restricting viral replication and spread (62). Our *in vitro* results show that CHIKV replication is supported by IL-17A, which may be due to the decrease in the production of IFN-α2. We further showed that *Ifn-α2* was upregulated in *Il17a^−/−^* and *Il17ra^−/−^* mice compared to WT mice after CHIKV infection, which may be the reason for lowering the level of CHIKV after infection. Our results further suggest that IL-17A, but not IL-17F, specifically inhibits the expression of IFN-α2 during CHIKV, but not WNV or ZIKV infection (**Figure 4** and **Supplementary Figure 2**). Both IL-17A and IL-17F belong to the same cytokine family and bind to the same receptors, but their specificity and binding affinity to the receptors differ. This might account for the differences observed in the inhibition of IFN-α2 and the replication of CHIKV in the presence of IL-17A and IL-17F. Alphaviruses are known for their antiviral counter defense strategies during infection, the C-terminal domain of CHIKV nsP2 specifically inhibits IFN response by promoting the nuclear export of STAT1 (63). Further, alphaviruses have evolved mechanisms to obstruct antiviral responses by inhibiting specific signaling pathways and modulating the host cell shutoff by the inhibition of general transcription and/or translation (64, 65). These properties of CHIKV might be involved in the inhibition of IFN-α2 expression during CHIKV infection, but not during WNV and ZIKV infection.

In mice, the family of type I IFN consists of 14 IFN-α subtypes and single forms of IFN-β, IFN-ϵ, IFN-κ, and IFN-ω (66). In response to viral infection, host cell PRRs recognize the viral ligands and leads to the production of IFN-β and IFN-α4 through activation of IRF-3 (67–71). Through autocrine and paracrine signaling, these IFNs modulate the expression of various transcriptional factors and ultimately induce expression of diverse IFN subtypes and IFN stimulated genes (ISGs). One of the transcriptional regulators induced by these IFNs is IRF-7, which induces all other IFN-α subtypes in a positive feedback loop, thus amplifying the response (72–74). Thus, IRF-3, IRF-5, and IRF-7 are considered to be the master regulators of type I IFN induction and ISG expression. The combined effect of these three transcription factors has been shown to coordinately regulate IFN response during WNV infection (75). In lethal CHIKV infection, the survival time of *Irf3^−/−^ Irf7^−/−^* double knockout mice are longer than *Ifnar1^−/−^* mice (7), thus speculating the role of additional transcription factors, such as IRF-5, contributing to the IFN response after CHIKV infection. Here, we show that IRF-3, IRF-5, and IRF-7 are inhibited by IL-17A at the mRNA levels during CHIKV infection; however, the phosphorylation of IRF-3 was not detectable by immunoblotting, suggesting IRF-3 may not play an essential role mediating IL-17A–IFN-α2 pathway. Instead, the expression and phosphorylation of *Irf-5* and *Irf-7* are inhibited by IL-17A, indicating that IL-17A signaling inhibits IFN-α2 mainly by affecting IRF-5 and IRF-7 mediated pathways (**Figure 5**). Type I IFNs bind to their receptors and initiate the JAK-STAT signaling pathways and induce the production of the ISGs that are critical to control viral replication. Our results show the transcript levels of *Isg-49* and *Mx1* decrease in the presence of rIL-17A while increase in the presence of IL-17A neutralizing antibody during CHIKV infection, thus suggesting IL-17A directly or indirectly inhibits the ISG-49 and Mx1-mediated antiviral responses against CHIKV replication.

Different IFN types may exert different functions during CHIKV infection, IFN-α limits early viral replication and dissemination; while IFN-β modulates neutrophil-mediated inflammation (62). Among IFN-α subtypes, IFN-α2 can be induced by Herpes Simplex Virus, Respiratory Syncytial Virus, and Newcastle Disease Virus (76). It has antiviral effects against influenza A virus and human metapneumovirus (76–78). We found that IL-17A inhibits the expression of IFN-α2 during CHIKV infection in a variety of cell types. The level of IFN-α2 was decreased and the viral replication was increased during CHIKV infection in the presence of rIL-17A. Our results also indicate only IFN-α2, but not the other 10 tested IFN-α subtypes is inhibited by IL-17A. Therefore, these results suggest that the increase of the production of IFN-α2 in the absence of IL-17A signaling may contribute to the reduced viral burden in the blood and footpad during CHIKV infection. Supporting this hypothesis, we showed that the level of CHIKV is less in the infected cells in the presence of recombinant IFNA2. Consistent with the previous research that has demonstrated that type I IFN signaling is essential to mitigate CHIKV-induced pathogenicity in mice (9, 62). Furthermore, we found that the infiltration of neutrophils into the footpads of *Il17ra^−/−^* mice was significantly reduced compared to WT mice on day 6 p.i. when the second peak of footpad swelling occurred. Neutrophils have been shown to be a major player contributing to CHIKV-induced inflammation and tissue damage in joints (79), which may be due to the increased level of inflammatory mediators, such as CXCL1, CXCL2, granulocyte colony-stimulating factor (G-CSF), IL-1β, and decreasing the level of anti-inflammatory macrophages. The lower viral burdens in the early phase of CHIKV infection seen in *Il17a^−/−^* and *Il17ra^−/−^* mice, may cause reduced levels of these inflammatory mediators, thus leading to a less severe footpad swelling on day 6 p.i.

In conclusion, our study discovers a novel role of IL-17A in inhibiting IFN-α2 production during CHIKV infection. Further studies are warranted to dissect the mechanism by which IL-17A regulates CHIKV-induced IFN-α2 expression, which may have broad implications in the understanding of IL-17A regulated immunity and development of novel IL-17A–based therapeutic strategy.

## Data Availability Statement

The raw data supporting the conclusions of this article will be made available by the authors, without undue reservation.

## Ethics Statement

The animal study was reviewed and approved by The Institutional Animal Care and Use Committees at The University of Southern Mississippi (USM).

## Author Contributions

FB conceived the experiments. BN, DA, FN, and GG-F conducted the experiments and analyzed the results. FB and BN wrote the manuscript. AF provided experimental materials. All authors contributed to the article and approved the submitted version.

## Funding

This work was supported in part by the National Institute of Allergy and Infectious Diseases of the National Institutes of Health R15AI35893 (FB). The authors also thank the Mississippi INBRE (funded by the National Institute of General Medical Sciences P20 GM103476) for use of the research facility. The funders had no role in study design, data collection and analysis, decision to publish, or preparation of the manuscript.

## Conflict of Interest

The authors declare that the research was conducted in the absence of any commercial or financial relationships that could be construed as a potential conflict of interest.

## References

[1] Ross RW The Newala epidemic. III. The virus: isolation, pathogenic properties and relationship to the epidemic. J Hyg (Lond) (1956) 54(2):177–91. 10.1017/S0022172400044442 PMC2218030 13346078

[2] Wahid B Ali A Rafique S Idrees M Global expansion of chikungunya virus: mapping the 64-year history. Int J Infect Dis (2017) 58:69–76. 10.1016/j.ijid.2017.03.006 28288924

[3] Silva LA Dermody TS Chikungunya virus: epidemiology, replication, disease mechanisms and prospective intervention strategies. J Clin Invest (2017) 127(3):737–49. 10.1172/JCI84417 PMC5330729 28248203

[4] Sourisseau M Schilte C Casartelli N Trouillet C Guivel-Benhassine F Rudnicka D Characterization of reemerging chikungunya virus. PloS Pathog (2007) 3(6):e89. 10.1371/journal.ppat.0030089 17604450 PMC1904475

[5] Kam YW Ong EK Renia L Tong JC Ng LF Immuno-biology of chikungunya and implications for disease intervention. Microbes Infect (2009) 11(14-15):1186–96. 10.1016/j.micinf.2009.09.003 19737625

[6] Chow A Her Z Ong EKS Chen JM Dimatatac F Kwek DJC Persistent arthralgia induced by chikungunya virus infection is associated with Interleukin-6 and Granulocyte macrophage colony-stimulating factor. J Infect Dis (2011) 203(2):149–57. 10.1093/infdis/jiq042 PMC3071069 21288813

[7] Schilte C Couderc T Chretien F Sourisseau M Gangneux N Guivel-Benhassine F Type I IFN controls chikungunya virus via its action on nonhematopoietic cells. J Exp Med (2010) 207(2):429–42. 10.1084/jem.20090851 PMC2822618 20123960

[8] Schilte C Buckwalter MR Laird ME Diamond MS Schwartz O Albert ML Cutting edge: independent roles of IRF-3 and IRF-7 in hematopoietic nonhematopoietic cells during host response to chikungunya infection. J Immunol (2012) 188(7):2967–71. 10.4049/jimmunol.1103185 22371392

[9] Rudd PA Wilson J Gardner J Larcher T Babarit C Le TT Interferon response factors 3 and 7 protect against Chikungunya virus hemorrhagic fever and shock. J Virol (2012) 86(18):9888–98. 10.1128/JVI.00956-12 PMC3446587 22761364

[10] McCarthy MK Reynoso GV Winkler ES Diamond MS Hickman HD Morrison TE MyD88-dependent influx of monocytes and neutrophils impairs lymph node B cell responses to chikungunya virus infection via Irf5, Nos2 ans Nox2. PloS Pathog (2020) 16(1):e1008292. 10.1371/journal.ppat.1008292 31999809 PMC7012455

[11] Heller E Enhancement of chikungunya virus replication and inhibition of interferon production by actinomycin D. Virology (1963) 21(4):652–6. 10.1016/0042-6822(63)90239-3 14100616

[12] Friedman RM Role of Interferon in viral interference. Nature (1964) 201:848–9. 10.1038/201848a0 14161240

[13] Wagner RR Inhibition of Interferon biosynthesis by actinomycin D. Nature (1964) 204:49–51. 10.1038/204049a0 14240110

[14] Li X Bechara R Zhao J McGeachy MJ Gaffen SL IL-17 receptor-based signaling and implications for disease. Nat Immunol (2019) 20:1594–602. 10.1038/s41590-019-0514-y PMC6943935 31745337

[15] McFarland HF Martin R Multiple sclerosis: a complicated picture of autoimmunity. Nat Immunol (2007) 8:913–9. 10.1038/ni1507 17712344

[16] Zepp J Wu L Li X IL-17 receptor signaling and T helper 17-mediated autoimmune demyelinating diseases. Trends Immunol (2011) 32:232–9. 10.1016/j.it.2011.02.007 PMC3329781 21493143

[17] van den Berg WB Miossec P IL-17 as a future therapeutic target for rheumatoid arthritis. Nat Rev Rheumatol (2009) 5:549–53. 10.1038/nrrheum.2009.179 19798029

[18] Raychaudhuri SP Role of IL-17 in psoriasis and psoriatic arthritis. Clin Rev Allergy Immunol (2013) 44:183–93. 10.1007/s12016-012-8307-1 22362575

[19] Newcomb DC Peebles RS Jr Th17-mediated inflammation in asthma. Curr Opin Immunol (2013) 25:755–60. 10.1016/j.coi.2013.08.002 PMC3855890 24035139

[20] Siakavellas SI Bamias G Role of the IL-23/IL-17 axis in Crohn’s disease. Discovery Med (2012) 14:253–62. 23114581

[21] Acharya D Wang P Paul AM Dai J Gate D Lowery JE Interleukin-17A promotes CD8+ T cell cytotoxicity to facilitate West Nile virus clearance. J Virol (2017) 91:e01529–16. 10.1128/JVI.01529-16 PMC5165211 27795421

[22] Bagri P Anipindi VC Nguyen PV Vitali D Stampfli MR Kaushic C Novel role for interleukin-17 in enhancing type 1 helper T cell immunity in the female genital tract following mucosal Herpes Simplex Virus 2 vaccination. J Virol (2017) 91(23):e01234–17. 10.1128/JVI.01234-17 PMC5686749 28956763

[23] Hou L Jie Z Desai M Liang Y Soong L Wang T Early IL-17 production by intrahepatic T cells is important for adaptive immune responses in viral hepatitis. J Immunol (2013) 190(2):621–9. 10.4049/jimmunol.1201970 PMC3538895 23233727

[24] Jie Z Liang Y Hou L Dong C Iwakura Y Soong L Intrahepatic innate lymphoid cells secrete IL-17A and IL-17F that are crucial for T cell priming in viral infection. J Immunol (2014) 192(7):3289–300. 10.4049/jimmunol.1303281 PMC3967589 24600029

[25] Yuan J Yu M Lin QW Cao AL Yu X Dong JH Th17 cells contribute to viral replication in Coxsackievirus B3-induced acute viral myocarditis. J Immunol (2010) 185(7):4004–10. 10.4049/jimmunol.1001718 20802148

[26] Hou W Kang HS Sim BS Th17 cells enhance viral persistence and inhibit T cell cytotoxicity in a model of chronic virus infection. J Exp Med (2009) 206(2):313–28. 10.1084/jem.20082030 PMC2646583 19204109

[27] Hou W Jin YH Kang HS Kim BS Interleukin-6 (IL-6) and IL-17 synergistically promote viral persistence by inhibiting cellular apoptosis and cytotoxic T cell function. J Virol (2014) 88(15):8479–89. 10.1128/JVI.00724-14 PMC4135960 24829345

[28] Cavalcanti NG MeloVilar K Duarte ALBP de Melo Rego MJB Pereira MC da Rocha Pitta I IL-27 in patients with Chikungunya fever: a possible chronicity biomarker? Acta Trop (2019) 196:48–51. 10.1016/j.actatropica.2019.05.005 31075222

[29] Lubberts E The IL-23-IL-17 axis in inflammatory arthritis. Nat Rev Rheumatol (2015) 11:415–29. 10.1038/nrrheum.2015.53 25907700

[30] Bai F Town T Qian F Wang P Kamanaka M Connolly TM IL-10 signaling blockade controls murine West Nile Virus infection. PloS Pathog (2009) 5(10):e1000610. 10.1371/journal.ppat.1000610 19816558 PMC2749443

[31] Bai F Wang T Pal U Bao F Gould LH Fikrig E Use of RNA interference to prevent lethal murine West Nile Virus infection. J Infect Dis (2005) 191:1148–54. 10.1086/428507 15747251

[32] Acharya D Paul AM Anderson JF Huang F Bai F Loss of glycosaminoglycan receptor binding after mosquito cell passage reduces chikungunya virus infectivity. PloS Negl Trop Dis (2015) 9(10):e0004139. 10.1371/journal.pntd.0004139 26484530 PMC4615622

[33] Paul AM Acharya D Duty L Thompson EA Linda Le Stokic DS Osteopontin facilitates West Nile Virus neuroinvasion via neutrophil “Trojan horse” transport. Sci Rep (2017) 7:4722. 10.1038/s41598-017-04839-7 28680095 PMC5498593

[34] Gardner J Anraku I Le TT Larcher T Major L Roques P Chikungunya virus arthritis in adult wild-type mice. J Virol (2010) 84:8021–32. 10.1128/JVI.02603-09 PMC2916516 20519386

[35] Teng TS Foo SS Simamarta D Lum FM Teo TH Lulla A Viperin restricts chikungunya virus replication and pathology. J Clin Invest (2012) 122(12):4447–60. 10.1172/JCI63120 PMC3533538 23160199

[36] Akitsu A Iwakura Y Isolation of Joint-Infiltrating Cells. Bio-protocol (2016) 6(17):e1911. 10.21769/BioProtoc.1911

[37] Teo TH Lum FM Claser C Lulla V Lulla A Mertis A A pathogenic role for CD4+ T cells during chikungunya virus infection in mice. J Immunol (2013) 190(1):259–69. 10.4049/jimmunol.1202177 23209328

[38] Morrison TE Oko L Montgomery SA Whitmore AC Lotstein AR Gunn BM A mouse model of chikungunya virus-induced musculoskeletal inflammatory disease: evidence of arthritis, tenosynovitis, myositis, and persistence. Am J Pathol (2011) 178:32–40. 10.1016/j.ajpath.2010.11.018 21224040 PMC3069999

[39] Genin P Lin R Hiscott J Civas A Differential regulation of human interferon A gene expression by interferon regulatory factors 3 and 7. Mol Cell Biol (2009) 29(12):3435–50. 10.1128/MCB.01805-08 PMC2698742 19349300

[40] Kotake S Udagawa N Takahashi N Matsuzaki K Itoh K Ishiyama S IL-17 in synovial fluids from patients with rheumatoid arthritis is a potent stimulator of osteoclastogenesis. J Clin Invest (1999) 103:1345–52. 10.1172/JCI5703 PMC408356 10225978

[41] van Bezooijen RL Farih-Sips HCM Papapoulos SE Lowik CWGM Interleukin-17: A new bone acting cytokine *in vitro* . J Bone Min Res (1999) 14:1513–23. 10.1359/jbmr.1999.14.9.1513 10469279

[42] Wong CK Ho CY Li EK Lam CW Elevation of proinflammatory cytokine (IL-18, IL-17, IL-12) and Th2 cytokine (IL-4) concentrations in patients with systemic lupus erythematosus. Lupus (2000) 9:583–93. 10.1191/096120300678828703 11035433

[43] Fujino S Andoh A Bamba S Ogawa A Hata K Araki Y Increased expression of interleukin 17 in inflammatory bowel disease. Gut (2003) 52:65–70. 10.1136/gut.52.1.65 12477762 PMC1773503

[44] Yen D Cheung J Scheeres H Poulet F McClanahan T Mckenzie B IL-23 is essential for T-cell mediated colitis and promotes inflammation *via* IL-17 and IL-6. J Clin Invest (2006) 116:1310–6. 10.1172/JCI21404 PMC1451201 16670770

[45] Albanesi C Cavani A Girolomoni G IL-17 is produced by nickel-specific T lymphocytes and regulates ICAM-1 expression and chemokine production in human keratinocytes: Synergistic or antagonistic effects with IFN-g and TNF-a. J Immunol (1999) 162:492–502. 10.1016/S0923-1811(98)84061-9 9886425

[46] Chabaud M Fossiez F Taupin JL Miossec P Enhancing effect of IL-17 on IL-1-induced IL-6 and leukemia inhibitory factor production by rheumatoid arthritis, synoviocytes and its regulation by Th2 cytokines. J Immunol (1998) 161:409–14. 9647250

[47] Miossec P Interleukin-17 in rheumatoid arthritis: If T cells were to contribute to inflammation and destruction through synergy. Arthritis Rheum (2003) 48:594–601. 10.1002/art.10816 12632409

[48] Shen F Ruddy MJ Plamondon P Gaffen SL Cytokines link osteoblasts and inflammation: Microarray analysis of interleukin-17- and TNF-alpha-induced genes in bone cells. J Leukoc Biol (2005) 77:388–99. 10.1189/jlb.0904490 15591425

[49] Lubberts E Koenders MI van den Berg WB The role of T cell interleukin-17 in conducting destructive arthritis: Lessons from animal models. Arthritis Res Ther (2005) 7:29–37. 10.1186/ar1478 15642151 PMC1064899

[50] Dong C Nurieva RI Regulation of immune and autoimmune responses by ICOS. J Autoimmun (2003) 21:255–60. 10.1016/S0896-8411(03)00119-7 14599850

[51] Gaffen SL Interleukin-17: A unique inflammatory cytokine with roles in bone biology and arthritis. Arth Res Ther (2004) 6:240–7. 10.1186/ar1444 PMC1064872 15535837

[52] Kolls JK Linden A Interleukin-17 family members and inflammation. Immunity (2004) 21:467–76. 10.1016/j.immuni.2004.08.018 15485625

[53] Koenders MI Kolls JK Oppers-Walgreen B van den Bersselaar L Joosten LA Schurr JR Interleukin-17 receptor deficiency results in impaired synovial expression of interleukin-1 and matrix metalloproteinases 3, 9, and 13 and prevents cartilage destruction during chronic reactivated streptococcal cell wall-induced arthritis. Arthritis Rheum (2005) 52:3239–47. 10.1002/art.21342 16200598

[54] Fossiez F Djossou O Chomarat P Flores-Romo L Ait-Yahia S Maat C T cell interleukin-17 induces stromal cells to produce proinflammatory and hematopoietic cytokines. J Exp Med (1996) 183:2593–603. 10.1084/jem.183.6.2593 PMC2192621 8676080

[55] Yao Z Fanslow WC Seldin MF Rousseau A-M Painter SL Comeau MR Herpesvirus Saimiri encodes a new cytokine, IL-17, which binds to a novel cytokine receptor. Immunity (1995) 3:811–21. 10.1016/1074-7613(95)90070-5 8777726

[56] Katz Y Nadiv O Beer Y Interleukin-17 enhances tumor necrosis factor alpha-induced synthesis of interleukin 1, 6, and 8 in skin and synovial fibroblasts: a possible role as a ‘fine-tuning cytokine’ in inflammation processes. Arthritis Rheum (2001) 44:2176–84. 10.1002/1529-0131(200109)44:9<2176::AID-ART371>3.0.CO;2-4 11592383

[57] Teng TS Kam YW Lee B Hapuarachchi HC Wimal A Ng LC A systematic meta-analysis of immune signatures in patients with acute chikungunya virus infection. J Infect Dis (2015) 211(12):1925–35. 10.1093/infdis/jiv049 PMC4442625 25635123

[58] Couderc T Chretien F Schilte C Disson O Brigitte M Guivel-Benhassine F A mouse model for Chikungunya: Young age and inefficient type-I interferon signaling are risk factors for severe disease. PloS Pathog (2008) 4(2):e29. 10.1371/journal.ppat.0040029 18282093 PMC2242832

[59] Ryman KD Klimstra WB Nguyen KB Biron CA Johnston RE Alpha/beta interferon protects adult mice from fatal Sindbis virus infection and is an important determinant of cell and tissue tropism. J Virol (2000) 74:3366–78. 10.1128/JVI.74.7.3366-3378.2000 PMC111838 10708454

[60] Trgovcich J Aronson JF Johnston RE Fatal Sindbis virus infection of neonatal mice in the absence of encephalitis. Virology (1996) 224:73–83. 10.1006/viro.1996.0508 8862401

[61] Laurent P Le Roux K Grivard P Betil G Naze F Picard M Development of a sensitive real-time reverse transcriptase PCR assay with an internal control to detect and quantify Chikungunya virus. Clin Chem (2007) 53:1408–14. 10.1373/clinchem.2007.086595 17586592

[62] Cook LE Locke MC Young AR Monte K Hedberg ML Shimak RM Distinct roles of interferon alpha and beta in controlling chikungunya virus replication and modulating neutrophil-mediated inflammation. J Virol (2019) 94(1):e00841-19. 10.1128/JVI.00841-19 31619554 PMC6912113

[63] Goertz GP McNally KL Robertson SJ Best SM Pijlman GP Fros JJ The methyltransferase-like domain of chikungunya virus nsP2 inhibits the Interferon response by promoting the nuclear export of STAT1. J Virol (2018) 92(17):e01008–18. 10.1128/JVI.01008-18 PMC6096799 29925658

[64] Strauss JH Strauss EG The alphaviruses – Gene-expression, replication and evolution. Microbiol Rev (1994) 58:491–562. 10.1128/MMBR.58.3.491-562.1994 7968923 PMC372977

[65] Gorchakov R Frolova E Williams BR Rice CM Frolov I PKR-dependent and -independent mechanisms are involved in translational shutoff during Sindbis virus infection. J Virol (2004) 78:8455–67. 10.1128/JVI.78.16.8455-8467.2004 PMC479073 15280454

[66] Pesch VV Lanaya H Renauld JC Michiels T Characterization of the murine alpha interferon gene family. J Virol (2004) 78(15):8219–28. 10.1128/JVI.78.15.8219-8228.2004 PMC446145 15254193

[67] Lin R Heylbroeck C Pitha PM Hiscott J Virus-dependent phosphorylation of IRF-3 Transcription Factor regulates nuclear translocation, transactivation potential, and proteasome-mediated degradation. Mol Cell Biol (1998) 18:2986–96. 10.1128/MCB.18.5.2986 PMC110678 9566918

[68] Sato M Tanaka N Hata N Oda E Taniguchi T Involvement of the IRF family transcription factor IRF-3 in virus-induced activation of the IFN-β gene. FEBS Lett (1998) 425:112–6. 10.1016/S0014-5793(98)00210-5 9541017

[69] Schafer SL Lin R Moore PA Hiscott J Pitha PM Regulation of type I Interferon gene expression by interferon regulatory factor-3. J Biol Chem (1998) 273:2714–20. 10.1074/jbc.273.5.2714 9446577

[70] Wathelet MG Lin CH Parekh BS Ronco LV Howley PM Maniatis T Virus infection induces the assembly of coordinately activated transcription factors on the IFN-β enhancer *in vivo* . Mol Cell (1998) 1:507–18. 10.1016/S1097-2765(00)80051-9 9660935

[71] Yoneyama M Suhara W Fukuhara Y Fukuda M Nishida E Fujita T Direct triggering of the type I interferon system by virus infection: activation of a transcription factor complex containing IRF-3 and CBP/p300. EMBO J (1998) 17:1087–95. 10.1093/emboj/17.4.1087 PMC1170457 9463386

[72] Marie I Durbin JE Levy DE Differential viral induction of distinct interferon-alpha genes by positive feedback through interferon regulatory factor-7. EMBO J (1998) 17:6660–9. 10.1093/emboj/17.22.6660 PMC1171011 9822609

[73] Honda K Takaoka A Taniguchi T Type I interferon gene induction by the interferon regulatory factor family of transcription factors. Immunity (2006) 25:349–60. 10.1016/j.immuni.2006.08.009 16979567

[74] Sato M Hata N Asagiri M Nakaya T Taniguchi T Tamaka N Positive feedback regulation of type I IFN genes by the IFN-inducible transcription factor IRF-7. FEBS Lett (1998) 441:106110. 10.1016/S0014-5793(98)01514-2 9877175

[75] Lazear HM Lancaster A Wilkins C Suthar MS Huang A Vick SC IRF-3, IRF-5, and IRF-7 coordinately regulate the type I IFN response in myeloid dendritic cells downstream of MAVS signaling. PloS Pathog (2013) 9(1):e1003118. 10.1371/journal.ppat.1003118 23300459 PMC3536698

[76] Loseke S Grage-Griebenow E Wagner A Gehlhar K Bufe A Differential expression of IFN-alpha subtypes in human PBMC: evaluation of novel real-time PCR assays. J Immunol Methods (2003) 276:207–22. 10.1016/S0022-1759(03)00072-3 12738374

[77] Moll HP Maier T Zommer A Lavoie T Brostjan C The differential activity of interferon-alpha subtypes is consistent among distinct target genes and cell types. Cytokine (2011) 53:52–9. 10.1016/j.cyto.2010.09.006 PMC3020287 20943413

[78] Scagnolari C Trombetti S Selvaggi C Carbone T Monteleone K Spano L In vitro sensitivity of human metapneumovirus to type I interferons. Viral Immunol (2011) 24:159–64. 10.1089/vim.2010.0073 21449726

[79] Poo YS Nakaya H Gardner J Larcher T Schroder WA Le TT CCR2 deficiency promotes exacerbated chronic erosive neutrophil-dominated chikungunya virus arthritis. J Virol (2014) 88(12):6862–72. 10.1128/JVI.03364-13 PMC4054367 24696480

