# Supplementary material for: Interleukin-17A Facilitates Chikungunya Virus Infection by Inhibiting IFN-α2 Expression
Source: Front Immunol. 2020 Nov 16;11:588382. doi: 10.3389/fimmu.2020.588382 (PMC7701120; doi:10.3389/fimmu.2020.588382)
Supplement: Supplementary file 1 [file Table_1.docx]

**Supplementary Table 1: Primer sequences used in the study**

| Primer | Forward 5’-3’ | Reverse 5’-3’ |
| --- | --- | --- |
| mβ-actin | AGA GGG AAA TCG TGC GTG AC | CAA TAG TGA TGA TGA CCT GGC CGT |
| hβ-actin | GCG CGG CTA CAG CTT CA | TCT CCT TAA TGT CAC GCA CGA T |
| mIL 17a | TCT CCA CCG CAA TGA AGA CC | TTT CCC TCC GCA TTG ACA CA |
| hIL17A | GTG GACT CTG GGA GGC AAA GT | GAT CTC TTG CTG GAT GGG GA |
| CHIKV E1 | TCC GGG AAG CTG AGA TAG AA | ACG CCG GGT AGT TGA CTA TG |
| Ifn-α | AGG ACA GGA AGG ATT TTG GA | GCT GCT GAT GGA GGT CAT T |
| Ifn-β | CGT TCC TGC TGT GCT TCT CC | TCT TGG AGC TGG AGC TGC TT |
| Ifn-γ | CAT TGA AAG CCT AGA AAG TCT G | CTC ATG AAT GCA TCC TTT TTC G |
| mIRF-3 | CGT ACA TCT GGG TGC CTC TC | ACC TCG AAC TCC CAT TGT TCC |
| mIRF-5 | AAT ACC CCA CCA CCT TTT GA | TTG AGA TCC GGG TTT GAG AT |
| mIRF-7 | CTG GAG CCA TGG GTA TGC A | AAG CAC AAG CCG AGA CTG CT |
| mSTAT-1 | GCT GAA TTC CAT CGA GCT CAC TCA | AAA CTC GAG CTC AGC TGC CAG ACT TCC |
| Rig-I | TTG CTG AGT GCA ATC TCG TC | GTA TGC GGT GAA CCG TCT TT |
| Mda5 | CGA TCC GAA TGA TTG ATG CA | AGT TGG TCA TTG CAA CTG CT |
| Myd88 | CAC TCG CAG TTT GTT GGA TG | TCT GGA AGT CAC ATT CCT TGC |
| Tlr-3 | TTG TCT TCT GCA CGA ACC TG | CGC AAC GCA AGG ATT TTA TT |
| Tlr-9 | ACT GAG CAC CCC TGA TTC TA | AGA TTA GTC AGC GGC AGG AA |
| Il-12 | CCT TGC ATC TGG CGT CTA CA | TTC TCT GGC CGT CAC CA |
| Cxcl2 | GCG CCC AGA CAG AAG TCA TA | CAG TTA GCC TTG CCT TTG TTC A |
| Cxcr3 | AGC CCT CAC CTG CAT AGT TG | TTG AGG CGC TGA TCG TAG TT |
| Ifn-α1 | AGAGAAGAAACACAGCCCCTG | GAGATATGAGCAGAGAGGTACAGT |
| Ifn-α2 | TTAGGAAGCAAGGGGAGGGT | AGCATCACGAGGAAAGCACA |
| Ifn-α4 | TTGCTGGCAAGACTGAGTGA | GAGAAGAATTGCTCAAGATTGCTGA |
| Ifn-α5 | GAGCCCTGTCTTCCTCAGTT | TGATCGCTCAAGATTGCTGAAAC |
| Ifn-α6 | GGACGAGTGAGTCCTGAGAC | ATGGTGCAGATACAAAAGTGGC |
| Ifn-α7 | GGAGGTGGTCAGAGCAGAAAT | CAGTGTCCTAATCCTGGAGATGG |
| Ifn-α8 | TGGAGAGACCTCCCTGGACT | ATGGTGCAGATACAAAAGTGGCT |
| Ifn-α9 | CAGCAGATCCTGACCCTCTTC | CAGTTCCTTCATCCCGACC |
| Ifn-α12 | CACTGACCCTCACTGCTCAA | TGCAGAGAGACATGACATTGC |
| Ifn-α13 | ACTTGCTGGCAAGACTGAGC | TCAGTTTTGCTGAAACATCTAGGC |
| Ifn-α14 | GCAGATCCTGACCCTCTTCAC | AGGTGCCTGTATCTCTACCTGC |
| Mx1 | GGCAAGGCTTCCGAGTTCTTC | CAGGGTGTCGATGAGGTCAAT |
| Isg-49 | GCCGTTACAGGGAAATACTGG | CCTCAACATCGGGGCTCT |
