# Supplementary figures and images for: Interleukin-17A Facilitates Chikungunya Virus Infection by Inhibiting IFN-α2 Expression
Source: Front Immunol. 2020 Nov 16;11:588382. doi: 10.3389/fimmu.2020.588382 (PMC7701120; doi:10.3389/fimmu.2020.588382)

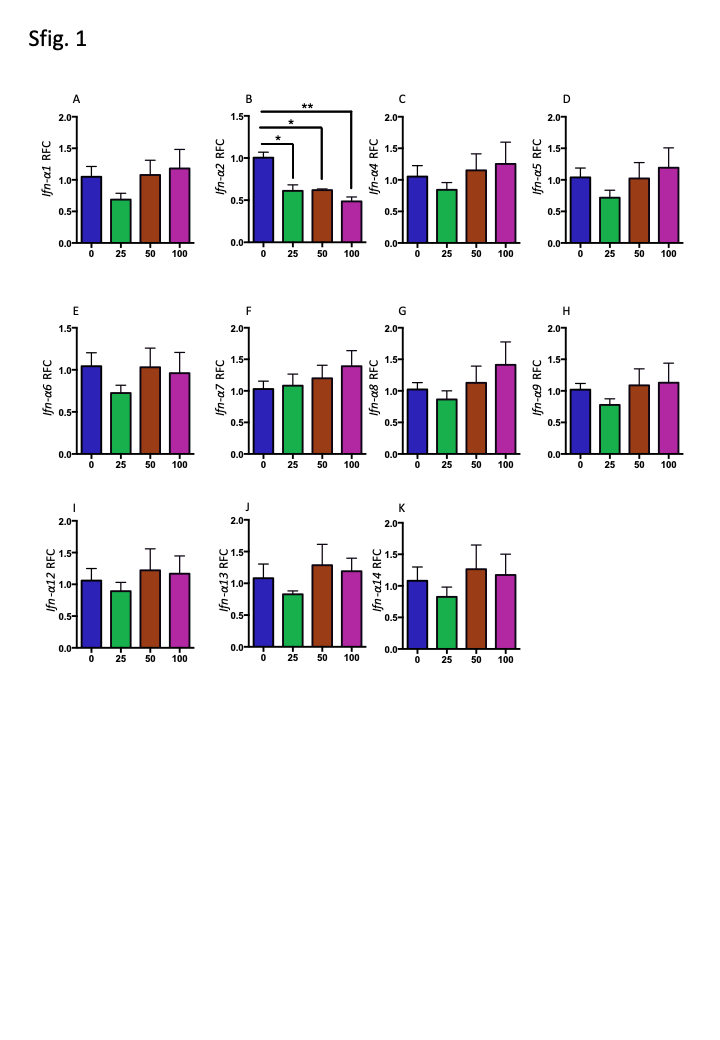

Supplement: Supplementary file 2 [file Image_1.tiff]

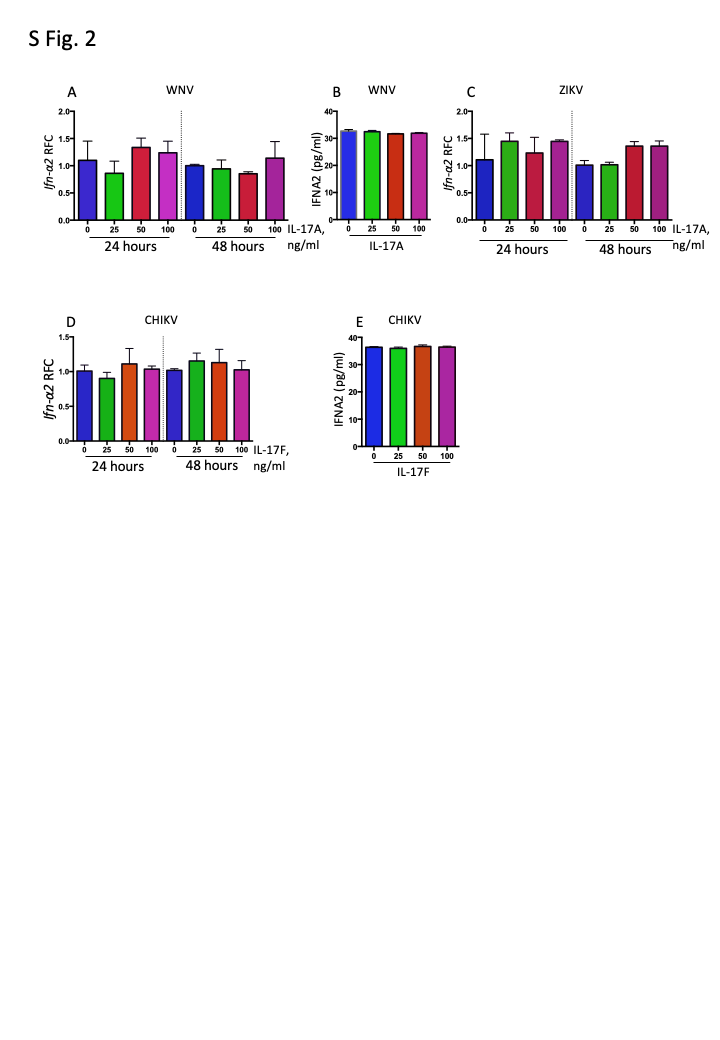

Supplement: Supplementary file 3 [file Image_2.tiff]
